# Alternatives to Antibiotics against *Mycobacterium abscessus*

**DOI:** 10.3390/antibiotics11101322

**Published:** 2022-09-28

**Authors:** Antonio Broncano-Lavado, Abrar Senhaji-Kacha, Guillermo Santamaría-Corral, Jaime Esteban, Meritxell García-Quintanilla

**Affiliations:** 1 Department of Clinical Microbiology, IIS-Fundación Jiménez Díaz, Av. Reyes Católicos, 2, 28040 Madrid, Spain; 2 CIBERINFEC—Infectious Diseases CIBER, Madrid, Spain

**Keywords:** *Mycobacterium abscessus*, nontuberculous Mycobacteria (NTM), alternative therapies, host modulation therapy, stem cells, phototherapy, antibiofilm therapy, phage therapy, nanoparticles, vaccines, antimicrobial peptides

## Abstract

*Mycobacterium abscessus* complex is extremely difficult to treat. Intrinsic and acquired bacterial resistance makes this species one of the most challenging pathogens and treatments last from months to years, associated with potential risky antibiotic toxicity and a high number of failures. Nonantibiotic antimicrobial agents against this microorganism have recently been studied so as to offer an alternative to current drugs. This review summarizes recent research on different strategies such as host modulation using stem cells, photodynamic therapy, antibiofilm therapy, phage therapy, nanoparticles, vaccines and antimicrobial peptides against *M. abscessus* both in vitro and in vivo.

## 1. Introduction

*Mycobacterium abscessus* is a rapidly growing nontuberculous mycobacteria (NTM) ubiquitous in the environment that is found in infections as an opportunistic pathogen. Although its taxonomy remains under debate, a widespread taxonomic classification suggests the differentiation of this species into three subspecies [1]: *M. abscessus* subsp. *abscessus*, *M. abscessus* subsp. *bolletii*, and *M. abscessus* subsp. *massiliense*. Infections caused by NTM are usually chronic and associated with immunological deficiencies, such as cystic fibrosis (CF). Morbidity and mortality of CF patients is due to both infections and unusual immune response [2]. Persistent infections in CF patients after lung transplant can result in significant morbidity and mortality [3]. NTM can cause a broad range of infections including pulmonary infections, systemic and/or disseminated infections, and skin and soft-tissue infections [4]. *M. abscessus* complex (MABC) is the most frequent isolate among rapidly growing NTM [5] and has been isolated in Asia (16%), Oceania (12%), America (8.9%) and Europe (2.9%) [6], with increasing rates of incidence [7,8,9].

Different virulence factors have been described in MABC, those include ESX-4 type VII secretion system (mandatory to survive and replicate into amoeba) [10], overexpression of GroEL-ES and Hsp (involved in shock and oxidative stress and necessary for intracellular growth in macrophages) [11], glycopeptidolipids (GPLs) extracellularly covering the cell wall (conferring smooth or rough morphotype, which results in increased virulence by forming a serpentine cording) [12,13], and MmpL permeases (involved in GPL transport or in glycosyl diacylated nonadecyl diol production) [14,15], among others.

*M. abscessus* colonizes the human body as a smooth variant and survives intracellularly into the phagosome of macrophages and neutrophils modulating cytokine expression to induce a granuloma formation [4]. Bacteria can remain in granulomas for several years or decades without symptoms since smooth variants lead to phagosome maturation blockage [4,16]. GPLs of smooth colonies are highly immunogenic, and adaptative immune cells are recruited to the site of infection. Unknown factors induce a morphological change into a rough variant and GLP-lack of rough variants facilitates bacterial aggregation forming extracellular cords whose large size prevents bacterial phagocytosis. Rough colonies induce a proinflammatory response as part of a severe infection resulting in massive tissue destruction [17]. Different in vivo models have shown that rough morphotypes are more virulent than smooth variants [18,19,20].

MABC is extremely difficult to deal with. Intrinsic and acquired bacterial resistance makes *M. abscessus* arguably the most challenging species of mycobacteria and treatments last from months to years associated with potential risky antibiotic toxicity and a high number of failures. 

Treatment of MABC pulmonary disease according to the British Thoracic Society encompasses an initial treatment phase with administration of various antibiotics including intravenous antibiotics (amikacin, tigecycline, and imipenem) combined with an oral macrolide (clarithromycin or azithromycin) for clinical isolates susceptible to macrolides. Subspecies *massiliense* is sensitive to macrolides, subspecies *abscessus* and *bolletii* are usually resistant due to the presence of an inducible methylase *erm*(41) gene. The continuation phase of the treatment involves nebulised amikacin and an oral macrolide combined with one to three of the following oral antibiotics: linezolid, clofazimine, minocycline, co-trimoxazole, and moxifloxacin [21]. Bacterial eradication is rare and recurrence can take place with time [22]. Additional recommendations can be found in the official ATS/ERS/ESCMID/IDSA Clinical practice guideline [23].

Some enzymes involved in antibiotic resistance in MABC have been reviewed recently [24]: methylase Erm(41) confers macrolide resistance through methylation of 23S rRNA. AAC(2′), Eis2 and APH(3″) add, respectively, acyl or phosphate groups to aminoglycosides preventing the binding of aminoglycoside to its ribosomal target. Eis2 can also modify other ribosome-targeting antibiotics like capreomycin. Arr_Mab ADP-ribosyltransferase is involved in the resistance to rifampicin and other rifamycin antibiotics by covalent modification and drug inactivation. MabTetX activates molecular oxygen to hydroxylate and destabilize tetracycline. Bla_Mab degrades b-lactams including extended-spectrum cephalosporins and carbapenems. 

In this scenario, alternative therapies are needed to handle these complicated infections. In this review, recent advances of nonantibiotic approaches that are currently being explored for prevention and treatment of *M. abscessus* infections are described, including host modulation therapy, phototherapy, antibiofilm therapy, phage therapy, nanoparticles, vaccines and anti-microbial peptides.

## 2. Host Modulation Therapy

This therapy investigates the possible therapeutic potential of mesenchymal stem cells (MSCs) in the macrophage system of *M. abscessus* infection due to their strong potential to modify the host immune response to an immunological condition [25].

MSCs are multipotent cells able to differentiate into multiple cell types such as osteoblasts, adipocytes, and chondroblasts [26]. Previous studies investigated the therapeutic potential of these multipotential cells for the treatment of diverse conditions such as bone diseases, graft-versus-host disease, autoimmune diseases, liver diseases, and cancer [26,27]. These investigations mostly focused on the immune-regulatory and tissue-reparative properties of MSCs as a result of immunosuppressive molecules and the expression of growth factors [26].

The immunosuppressive properties of MSCs have been well characterized and defined, but the immune-enhancing ability of MSCs have not. MSCs have exhibited antimicrobial activity against some bacterial pathogens, such as *Escherichia coli*, *Pseudomonas aeruginosa*, *Staphylococcus aureus* and *Streptococcus pneumoniae*, by secreting antimicrobial peptides and inducing host innate responses [28,29,30]. Some studies have demonstrated the beneficial effects of MSCs in sepsis models [31,32,33] and to clear extracellular *E. coli* [34,35]. 

Host susceptibility to the development of NTM disease has been related to low levels of Th1-associated cytokines and a delayed infiltration of innate cells into the lungs [25,36,37]. Elsewhere, it has been demonstrated that MSCs induce the recruitment of inflammatory cells in the lungs and the production of proinflammatory cytokines. These proinflammatory cytokines (such as IFN-γ, and TNF-α) increase the production of PGE2 [38], which increases the NO production in *M. abscessus*-infected macrophages [25,36,37]. MSCs constitutively produce PGE2 but under inflammatory conditions its production is augmented [39]. All these mechanisms contribute to the clearance of the bacterial pathogen.

Other studies have determined that the infection of macrophages with virulent H37Rv *M. tuberculosis* induces lipoxin A4 production and blocks PGE2, leading to the death of *M. tuberculosis*-infected macrophages [40,41,42]. Most of previous studies have focused on the role of PGE2 in relation to the type of cell death that occurs in infected macrophages. However, the effector role of PGE2 in the killing mechanisms against mycobacterial bacteria and the molecular mechanisms of PGE2 -induced NO production in macrophages remain poorly understood and need further investigation.

It is important to highlight that MSCs have been reported to accumulate in the lungs following intravenous administration, which is the main site of *Mycobacterium* infection. Interestingly, redifferentiated mucosal-associated invariant T (reMAIT) cells have been demonstrated to develop antimycobacterial activity against *M. abscessus* in murine models reducing bacterial load by liberating granulysins [43], as was shown for *M. tuberculosis* [44]. These granulysins were detected in murine sera, but neither IFN-γ nor TNF-α were detected. This suggests that granulysin is an effector molecule responsible for combating intracellular bacteria [45]. Due to the expression of CCR6, IL-12Rb2, IL-23R, RORC, IL-17, and IL-22, reMAIT cells could be characterized as a subset of Th or Tc17 cells or a novel family of lymphoid-tissue-inducer cells with an immunoregulatory function [46]. Granulysin is specific to humans and has no homolog in mice, so the use of reMAIT cells could elucidate the mechanism of host immune defense in humans [43].

Other studies have determined that CD271+ MSCs proportionate a protective intracellular environment in the host in which *M. tuberculosis* can survive and provides antibiotic protection [47]. Thus, endogenously and exogenously, MSC administration can act differently in response to *Mycobacterium* spp. depending on immunologic conditions. 

Although there are no clinical studies about MSC therapy in MAC infections, several clinical studies against *M. tuberculosis* infections using treatment based on MSCs have shown positive results [48,49]. Systemic transplantation of autologous MSCs into multidrug-resistant *M. tuberculosis* patients induced positive clinical effects [50] and a clinical trial of an autologous MSC therapy against multidrug-resistant *M. tuberculosis-* infected patients has been developed [49].

Taking into account the recommendations of Zhang et al. in their review about this therapy against *M. tuberculosis* infections [51], MSC-based MAC treatment should pay attention to some issues: (1) Depending on the different tissue sources and culture conditions in vitro, there are differences in the biological functions of MSCs [52,53], therefore it is important to select the MSCs from appropriate tissue sources and optimize the culture conditions in vitro. (2) Some clinical studies in *M. tuberculosis* have demonstrated that MSC treatment still has some side effects [54]; due to the lack of clinical trials, it cannot be fully proved that MSCs are safe as adjuvant therapy against *Mycobacterium* infections. (3) Mycobacteria can infect macrophages, alveolar epithelial cells, and MSCs depending on the lifestyle of host cells, which may be one of the reasons for *Mycobacterium* resistance. Accordingly, a better understanding of the relationship between *Mycobacterium* and host cells would be key to the treatment of these bacterial infections.

## 3. Photodynamic Therapy

Photodynamic therapy (PDT) was discovered more than a hundred years ago and it is used mainly for cancer treatment, ophthalmologic disorders and in dermatology. However, in recent years, the interest in the antimicrobial effects of PDT has increased and has been proposed as a therapy for a large variety of localized infections especially those that are drug resistant. In particular, PDT is able to sterilize infections using an aromatic molecule as a photosensitizer (PS) that reaches a high energy, excited state when it is irradiated with a visible or near infrared light [55]. Even though the exact mechanism of action of PDT is not clear, the reactive oxygen species produced in this combination seems to be the main reason for the bactericidal effect. These active oxygen substances can kill microorganisms by damaging various cellular components, through the photo-oxidation of nucleic acids, proteins and lipid membrane resulting in death of the targeted cell [56].

PDT has been successfully used in vitro and in vivo in several animal models of localized infections such as surface wounds, burns, oral sites, abscesses and otitis and is being clinically studied for other dermatological infections such as leishmaniasis [57].

In general, neutral, anionic or cationic PS molecules can efficiently kill Gram-positive bacteria, whereas only cationic PS or approaches that pass through the Gram-negative permeability barrier in combination with non-cationic PS are able to kill Gram-negative bacteria. In a recent study, tetra-cationic and anionic porphyrins were used as PSs for the photodynamic inactivation of rapidly growing mycobacteria strains. Two different charged porphyrin groups (cationic and anionic) were irradiated with white light at a fluence rate of 50 mW/cm^2^ over 90 min; it was shown that porphyrins with positive charge was the most effective PS causing a 100–fold reduction in the concentration of viable mycobacteria compared with anionic charged porphyrins. The authors concluded that the effectiveness of the PS for PDI studies against mycobacteria is strongly related to the porphyrin peripheral charge, and consequently with PS solubility in physiological media. Therefore, cationic photosensitizers might be promising anti-mycobacteria PDI agents with potential applications in medical clinical cases [58]. 

Although efficiency is very variable between molecules with different transition metal coordination compounds, a recent study has demonstrated the effectiveness of positively charged porphyrins at the Meta position (3-PtTPyP) under white-light illumination for 90 min used as PS in PDT against *M. abscessus*. Some of the factors contributing to the satisfactory activity of these PS against mycobacteria are ROS production, low aggregation in the solution, higher photostability and higher white-light dosage [3]. Another study assessed the effect of PDT using 5-aminolevulinic acid (ALA) as a PS against *M. abscessus* and its biofilm. Both were treated using different concentrations of ALA and irradiated with LED light (635 nm, 80 J/cm^2^). The experiments were conducted using three groups: ALA-only group, light-only group and negative control group. Results showed an important killing effect on the microorganism using ALA-PDT at concentrations higher than 50 μg/mL, and the effect increased with higher concentrations. The same effect was observed on *M. abscessus* biofilm, which also was enhanced with increasing ALA concentrations. Importantly, the susceptibility of *M. abscessus* to antibiotics was increased at sublethal doses and ALA-PDT due to the higher cell-wall permeability of the bacteria [59].

Guterres et al. evaluated the use of six water-soluble cationic porphyrins as PSs for the antimicrobial PDT against *M. abscessus*. Experiments were conducted with the same concentration of PS under white-light irradiation conditions over 90 min. The results showed that porphyrins 1 and 2 (M = 2H or ZnII ion) were the most effective and significantly reduced the concentration of viable mycobacteria, suggesting that success in treatment was dependent on the metal-center ion coordinated in the cationic porphyrin core [60]. 

A case report described a 74-year-old man with a persistent erythematous plaque affecting the occipital region of the scalp. A skin biopsy and a culture test revealed an atypical mycobacteriosis. A cycle of five sessions of conventional PDT was undertaken using the PS agent methyl aminolevulinate (MAL) left under occlusion for 3 h, then illuminated with a red light-emitting diode lamp (630 nm). MAL-PDT was performed once a month, at increasing dosages from 37 to 60 J/cm^2^ (irradiation time ranging from 8.5 to 14.5 min). The treatment was well tolerated by the patient and showed a progressive improvement, both clinically and dermoscopically from one session to another. A complete healing and resolution of the clinical case was achieved after the fifth session. The patient was followed up for one year, showing no signs of recurrence [61].

There are some advantages of PDT that makes this therapy attractive, such as the equal killing effectiveness regardless of antibiotic resistance, a broad antimicrobial spectrum, low host damage and side effects, inactivation of toxins and lack of PDT resistance. However, the main limitation is the restriction to topical use only.

## 4. Antibiofilm

The ability of *M. abscessus* to develop biofilms is related to its antimicrobial resistance. The rough morphotype of *M. abscessus* is one of hyperaggregation and forms biofilm-like aggregates, while smooth colonies can form biofilm aggregates [62]. The possibility of reducing the formation of these biological structures is a very promising therapeutic approach. In 2017, Rossi et al. studied the influence of essential oil and nanoemulsions of *Cymbopogon flexuosus* on the production of biofilms on standard strains of *M. fortuitum* and *M. abscessus* and on their planktonic cells. The nanoemulsions showed significant antibacterial activity. Importantly, both were able to prevent biofilm formation and de novo formation, demonstrating a high therapeutic potential against fast-growing mycobacterial infections [63]. Interestingly, García-Coca et al. determined the in vitro activity of *Methylobacterium* sp. in biofilm formation in fast-growing mycobacteria. For this purpose, suspensions of live *Methylobacterium* sp bacteria, autoclaved suspensions or sonicated extracts of this bacterium were added to pre-formed biofilm of reference strains of *M. abscessus*, *M. chelonae*, and *M. fortuitum* at different times. Results showed a reduction in biofilm thickness and surface area as well as a reduction in bacterial counts. In the case of *M. abscessus*, there was a very significant reduction after 72 h with the addition of the sonicated extract and with the autoclaved suspension [64].

More recently, Kolpen et al. studied the influence of oxygenation on biofilm formation and antibiotic sensitivity of MAC. The authors used 33 clinical isolates from 22 patients and a reference strain and studied their sensitivity to the following antibiotics: amikacin, azithromycin, cefoxitin, ciprofloxacin, clarithromycin, imipenem, kanamycin, linezolid, moxifloxacin, rifampicin, tigecycline and co-trimoxazole. Clinical isolates were cultured on Muller-Hinton in absence and presence of Tween 80. Results showed that disruption of *M. abscessus* aggregates improved sensitivity to amikacin, tigecycline, kanamycin, azithromycin, azithromycin, imipenem, cefoxitin and clarithromycin; and oxygenation improved clearance of aggregates with amikacin. They concluded that antibiotic sensitivity studies correlate poorly with patient treatment outcomes because the conditions of these tests do not mimic the hypoxic lung conditions of patients with cystic fibrosis, suggesting that oxygenation of these patients may help in chronic MAC infection [65].

The influence of complex sulfonamides and metals Au, Cd, Ag, Cu and Hg has also been investigated. *M. abscessus* and *M. fortuitum* were studied in vitro growing on polystyrene with different complex sulfonamides (sulfadiazine Au-Pᴓ3, sulfadiazine ᴓ2P-Au-Au-Pᴓ2, sulfamethoxazole Au-Pᴓ3, sulfamethoxazole ᴓ2P-Au-Au-Pᴓ2, sulfamethoxazole Au, sulfamethoxazole Ag, sulfamethoxazole Hg, sulfamethoxazole Cd and sulfamethoxazole Cu). Complex sulfonamides significantly reduced the growth of mycobacteria and decreased their ability to form biofilm by inhibiting the synthesis of c-di-GMP [66]. In 2022, Belardinelli et al. screened 30 2-aminoimidazole compounds with the aim of developing molecules with the ability to inhibit mycobacterial biofilm. These compounds demonstrated in vitro activity against *M. tuberculosis* and *M. smegmatis* and were subsequently screened against *M. abscessus*. Only the AB-2-29 compound showed antibiofilm activity against this pathogen with an IC50 (concentration required to inhibit 50% of biofilm formation) in the range of 12.5 to 25 μM. Curiously, the activity of AB-2-29 was enhanced by the addition of Zn to the culture medium [67]. 

This approach could be a promising alternative for lung or prosthetic-related infections, whose bacterial resistance is enhanced with biofilms. Nevertheless, the research in this field is scarce for mycobacteria and more studies are needed, especially in vivo studies, prior to clinical use.

## 5. Phage Therapy 

Bacteriophage therapy is an old therapy which uses lytic bacteriophages against bacteria. Bacteriophages against mycobacterial hosts are known as mycobacteriophages and all characterized mycobacteriophages are phages with double-stranded DNA genome (dsDNA) [68]. Currently, over 11,000 mycobacteriophages have been isolated, and 2000 of them have been sequenced [69]. 

There is an increasing interest in this alternative therapy and both in vitro and in vivo studies as well as case reports and even clinical trials have been performed against different pathogens, such as *Pseudomonas aeruginosa* or *Staphylococcus aureus*. However, the number of studies against mycobacteria is scarce. Mycobacteriophages have been explored in vitro and recent case reports involving NTM infections have been reported. One compassionate use of phage therapy against mycobacteria was the treatment of a 15-year-old patient suffering from CF with a disseminated *M. abscessus* infection after a bilateral lung transplantation. A three-phage cocktail was administered intravenously and well tolerated. Clinical improvement was achieved including sternal wound closure, better liver function and even resolution of infected skin nodules [70].

In another case, an 81-year-old man with non-CF bronchiectasis and refractory macrolide-resistant *M. abscessus* lung infection was treated using a three mycobacteriophage cocktail by intravenous route twice a day during six months in addition to a multidrug antibiotic combination. After one month with intravenous treatment, there was a decline in bacterial count from sputum but the treatment was rapidly ineffective due to anti-phage neutralizing antibodies and phage resistance to one phage. The treatment was discontinued due to lack of clinical effectiveness [71]. Less than three months after, the patient started with nebulized administration of phages during nine months to overcome serum neutralization and to increase phage delivery to the site of infection. Treatment started with the identical cocktail containing the same three mycobacteriophages used for intravenous route. Nebulized phage delivery was relatively safe, well tolerated by the patient, and effective in reducing sputum production. Four months post-nebulized phage delivery, benefits of the therapy began to decrease and administration changed after 7.5 months to a vibrating mesh-type nebulizer, which did not translate in to significant improvements. Post-treatment findings showed that phage resistance was not the reason behind the clinical failure: instead, IgA reactivity may have occurred as a response to previous intravenous administration. Neutralization appeared at months 7 and 8 contributing to the treatment limitation [72]. Antibody-mediated neutralization can appear after one or two months of treatment and may be a critical factor in phage therapy efficacy [73]. 

Another compassionate use of phages was administered to a 26-year-old male with treatment-refractory *M. abscessus* pulmonary infection and severe CF lung disease. Two engineered mycobacteriophages were manufactured to increase their ability to lyse *M. abscessus* and were specifically selected as the most effective against the specific bacterial isolate. Phages were administered intravenously twice a day for over a year and were well tolerated with no adverse reactions assigned to bacteriophage therapy. *M. abscessus* isolates presented genetic stability during the therapy. Nevertheless, anti-phage neutralizing antibodies titers increased with time for one of the phages but did not affect clinical improvement significantly. The patient was successfully transplanted on day 379 post-phage therapy and systemic cultures of the explanted lung were negative for *M. abscessus* [74].

Importantly, in a recent study, compassionate use of phages in twenty patients infected with NTM has been described [75]. Phages were administered intravenously, by aerosolization, or both, during an average of six months on a compassionate use basis. No adverse reactions were found and all patients received additional antimycobacterial treatment with at least two drugs based on prior drug susceptibility testing. Clinical improvement or microbiological responses were observed in 11 patients, including infants. From the 20 patients proposed for treatment, 17 suffered from *M. abscessus* infections (14 CF patients, one suffered from bronchiectasis, one from scleroderma and one from hypersensitivity pneumonitis). Among the 17 *M. abscessus* isolates, 12 were subspecies *abscessus* and 5 isolates were subspecies *massiliense*. In total, 8 out of 17 cases resulted in favorable or partially favorable outcomes, 5 patients showed inconclusive outcomes, and 4 patients had no response. No differences were observed relating to the administration route or type of infection. This study showed complete eradication in some infections and successful lung transplant in one patient. 

Bacteriophage therapy could become a promising personalized medicine in patients with prolonged or relapsing mycobacterial infections. However, there is a need for research to predict the success of cocktails from in vitro to in vivo, appearance of bacterial resistance, host neutralization and understanding around the best choice for administration.

## 6. Nanoparticles

The use of nanoparticles allows an improvement in the delivery of different molecules, and this strategy can be used as a novel therapy against multi-drug resistant bacteria. In 2011, the activity of nine series of dicarboxylic and tricarboxylic amphiphiles with one alkyl, two alkyl and cholestanyl tails was tested against various NTM, including *M. abscessus*. These dendritic amphiphiles had physicochemical characteristics that gave them adequate antimycobacterial activity with high mycelial critical concentrations and very low haemolytic activity [76]. Seven years later, Choi et al. studied the activities of different gallium compounds including Ga(NO_3_)_3_, GaCl_3_, gallium meso-tetraphenylporphyrin (GaTP) and gallium nanoparticles (GaNP). The authors determined the concentrations of these compounds in THP-1 macrophages and studied the growth of mycobacteria inside these macrophages as well as the biodistribution of Gallium in mice. These experiments showed that GaTP was more effective than Ga(NO_3_)_3_ and GaCl_3_. GaNP was able to penetrate into macrophages and exhibited a greater effect in inhibiting the bacterial growth. Gallium compounds inhibit the mechanisms of action of the siderophores and prevent the uptake and use of iron by the bacterial cell. In turn, the addition of exogenous iron and hemin reverses the action of gallium, allowing MABC to grow. Moreover, the mouse model showed that the distribution of these nanoparticles in the lung was more effective via peritoneal injection than via intramuscular injection [77].

More recently, in vitro studies about encapsulated clarithromycin in PLGA nanocapsules have been performed against different pathogens, including *M. abscessus*. The authors used smooth and rough strains of MAC. These strains were inoculated into mouse macrophage cell lines, in a murine wound model of infection and in a zebrafish model of infection. The clearance of *M. abscessus* exposed to the clarithromycin nanocapsules in the in vitro model was 70–80% higher than with the free drug. Furthermore, the in vivo model showed that the permeability in subepithelial tissues was higher than with the free drug [78]. Interestingly, Choi et al. conducted a new study of nanoparticles applied to *M. abscessus* and *M. avium*. In this work, the authors synthetized a beta-cyclodextrin, which contained gallium atoms and a gallium tetraphenylporphyrin. Both formulations were tested in an in vitro model of macrophage infection and in an in vivo model of lung infection in mice. Beta-cyclodextrin showed greater inhibition than the porphyrin compound against both mycobacteria in the in vitro model, with sustained release of gallium atoms for 15 days and effectivity in the mouse lung infection model [79]. Other authors tested nanosomes loaded with antibiotics (rifabutin and ciprofloxacin) and nanoparticles with the lignin-silver complex. These new formulations were tested in an in vitro model of human THP-1 macrophage infection using a reference strain of *M. abscessus*. Cytotoxicity was studied using the 3-(4,5-dimethylthiazol-2-yl)-2,5-diphenyltetrazolium bromide (MTT) and by measuring proinflammatory and anti-inflammatory cytokines in THP-1 macrophage cell culture. *M. abscessus* was susceptible to the nanosomes tested in THP-1 macrophages, suggesting that they were internalized into the macrophage cell and did not stimulate the secretion of proinflammatory cytokines. The authors concluded that nanosomes may represent a new therapeutic option by decreasing drug toxicity and improving efficacy [80].

The use of nanoparticles increases chemical stability of pharmacological agents and reduce side effects, making the strategy very attractive for use against resistant bacteria. However, the scaling and quality control of nanoparticles is challenging and stability is particularly complex in the case of nanomaterials.

## 7. Vaccines

Vaccination has been traditionally the nonantibiotic strategy with the highest rate of success and impact in the population. There are several vaccine candidates with different results against *M. abscessus*. In 2015, Le Moigne et al. used reverse genetics to identify phospholipase C (MA-PLC) as a target for vaccination against *M. abscessus* for CF patients. The authors used the plasmid coding for MA-PLC and formulated MA-PLC with a tetra-functional block copolymer 704. Smooth and rough strains were aerosolized in CF (F508) mice. Vaccination resulted in high antibody production against the smooth variants but not against the more hypervirulent rough variants [81]. In an ulterior study, Le Moigne et al. studied the virulence factor MgtC involved in increasing the lifespan of *M. abscessus* in macrophages. This factor was frequently detected in the serum of CF patients infected with *M. abscessus*. Transcriptional and translational upregulation of MgtC led to increase survival of the mycobacterium in the macrophage. The authors performed in vivo immunization in F508 FVB mice with CF using DNA sequence encoding for MgtC formulated in a tetrafunctional amphiphilic copolymer. Administration of this preparation to mice increased the antibody levels against the MgtC factor, leading to the conclusion that this may provide a basis for the development of new therapeutic options in CF patients [82].

Interestingly, Lee et al. (13) studied the *M. abscessusMAB1843* gene encoding a D-alanyl-D-alanine dipeptidase. This protein had the ability to interact with human dendritic cells and induce their maturation to stimulate the proliferation of TH1-specific T lymphocytes [83]. Importantly, the effect of Bacillus Calmette-Guerin (BCG) vaccine on immunity against *M. avium* and *M. abscessus* has also been analyzed. This vaccine is used in children in tuberculosis-endemic areas. The authors quantified by flow cytometry the T lymphocytes that cross-protected against tuberculosis and non-tuberculous mycobacteria, and then assessed the ability of these BCG-expanded T lymphocytes to inhibit the intracellular growth of *M. avium* and *M. abscessus*. Cross-immunity against NTM in BCG-immunised and tuberculosis-infected mice was also analyzed. The results showed that mononuclear cells, activated in individuals who had received the BCG vaccine and in individuals with a history of tuberculosis, were cross-reactive against *M. avium* and *M. abscessus*. Moreover, both BCG vaccination and tuberculosis infection in mice resulted in increased production of T cells cross-reactive against *M. avium* and *M. abscessus*, which inhibited the intracellular growth of these mycobacteria. The authors concluded that the BCG vaccine has the potential to prevent and treat NTM infections [84].

More recently, Le Moigne et al. isolated compounds from the surface of rough *M. abscessus* strains that are involved in the exacerbation of the inflammatory response caused by this mycobacterium. This inflammatory cascade is mediated by Toll-like receptors. These extracts contained lipoproteins and proteins that stimulate the action of TLR-2 receptors and were termed TLR2eF. TLR2eF fractions were administered to 1F508 and FVB mice in nebulized form and gave low protection against *M. abscessus*, but increased antibody production was detected when administered intravenously. TLR2eF fractions were also tested in patients with cystic fibrosis and antibodies were detected against these substances, indicating that immunological induction was taking place [85]. A computational study was carried out by Da et al. compiling 34 complete genome sequences of *M. abscessus* from the NCBI GenBank database; they performed a genomic analysis to determine the genetic diversity of these genomes and identified possible antigenic targets for the design of multi-epitope vaccines. The authors identified four potential antigenic targets and computationally designed a vaccine with multiple epitopes. They estimated the molecular dynamics by the ability to bind to the Toll-2 receptor. Their results were promising but still require in vivo studies to be properly validated [86]. 

Prophylactic vaccination represents an approach that could be highly effective for preventing MAC infections in risky patients such as CF patients. Epidemiological studies and precise risk factors for *M. abscessus* infections would help to address the target population of this therapy.

## 8. Antimicrobial Peptides

Antimicrobial peptides are key elements of the immune system of higher organisms, making them potential candidates for use as antibacterial agents. There are various candidates against *M. abscessus* to date (Table 1). A new peptide called Polydim-I, from the neotropical wasp Polybiadimorpha, was studied. This peptide had the ability to cleave the wall of *M. abscessus* and did not exhibit cytsotoxicity in animal cells. The use of Polydim-I in macrophages infected with different strains of *M. abscessus* reduced the bacterial load by 40–50%. This was followed by in vivo assays in mice in which a 0.8 to 1 log reduction was detected in murine liver, lung and spleen bacterial loads [87]. 

In another study, Silva et al. described the Polybia-MPII peptide from *Pseudopolybiavespiceps* and analyzed its activity against various opportunistic pathogens, including *M. abscessus*. The peptide was studied in an ex vivo mouse peritoneal macrophage assay and subsequently in an in vivo mouse wound model assay and was able to inhibit mycobacterial growth by 80% at a concentration of 12.5 µM but was unable to do so at a concentration of 6.25 µM. However, high haemolytic and cytotoxic activity was detected against red blood cells and macrophages in both humans and mice, being higher in the latter [88].

In 2017, a new antimicrobial peptide called NDBP-5.5 was isolated from scorpion venom. Its minimum inhibitory concentration (MIC) against *M. abscessus* was determined to be 200 µg, which did not induce haemolysis of human erythrocytes in the haemolytic assay performed. The action of this peptide was compared in mice with that of clarithromycin and achieved similar results, reducing the bacterial load by 70% [89]. In 2020, Da Silva et al. studied the action of a series of antimicrobial peptides against three clinical strains of *M. abscessus* from CF patients. The peptides studied were AP1, AP2, AP3 (D enantiomeric AP1), AP4, AP5 and AP6 (ATRA-1A). The activity of these peptides was evaluated in microdilution plates obtaining MICs from 1.6 to 50 µg [90]. 

Recently, between 2012 and 2016, a total of 13 antimycobacterial peptides (S5, S52, S6, S61, S62, S63, KLK, KLK1, KLK2, Pug-1, Pug-2, Pug-3 and Pug-4) were evaluated against 16 clinical isolates from Thailand and against the ATCC19977 strain. These peptides were tested in vitro and only 4 were effective (S61, S62, S63 and KLK1) providing MICs between 200–400 µg/mL. Interestingly, most of the clinical strains (10/16) were sensitive to all 4 peptides. In the haemolytic assay, S63 was the least toxic peptide and KLK1 the most toxic peptide to erythrocytes [91]. In 2021, Li et al. investigated the activity of the newly synthesised peptide RP557 alone and in combination with antibiotics clarithromycin, amikacin, cefoxitin and imipenem against *M. abscessus* in biofilm. The peptide alone showed moderate activity against *M. abscessus* in vitro; however, combining it with antibiotics significantly increased the sensitivity to antibiotics. Scanning electron microscopy showed that RP557 was able to inhibit biofilm production and staining showed an increase in bacterial killing. Transcriptome analysis determined that RP557 inhibited biofilm production and bacterial growth by interfering with nitrogen metabolism, fatty acids and peptidoglycan [92].

In another study, the antibacterial activity of NZX peptide was studied against *M. tuberculosis* and *M. abscessus*. The NZX peptide had the ability to inhibit the growth of *M. abscessus* in both in vitro and in vivo models [93]. Interestingly, a recent work developed a high-density peptide microarray consisting of 125,000 randomly synthesized peptides. These peptides were then screened against various strains of *M. abscessus*. Six of them showed activity in vitro against the smooth morphotype of *M. abscessus* (IC50 = 1.7 μM for all peptides) and very low activity against the rough morphotype of *M. abscessus* (IC50: 13–82 μM). ASU2056 and ASU2060 showed MICs of 32 and 8 μM against the smooth morphotype of *M. abscessus*. These peptides did not show haemolytic activity and were stable in human serum after pre-incubation in this medium for 24 h [94]. 

There is a lack of experiments to demonstrate safety of antimicrobial peptides for therapeutic use, mainly for systemic administration due to the resistance to proteolytic degradation in serum. Nevertheless, these molecules could be eligible for topical use. A challenge of this therapy is to develop analogs with lower toxicity and susceptible to proteolytic degradation.

## 9. Concluding Remarks

*M. abscessus* infections require novel approaches due to the limitations of current antibiotic treatments. Taking into account the relapses caused by this microorganism, it seems reasonable that alternative therapies may be used to eradicate this pathogen from patients. Depending on the case, one strategy or another could be recommended in the future. For instance, vaccination would be desirable for patients with pulmonary risk, such as CF patients, bronchiectasis or chronic obstructive pulmonary disease; host modulation using stem cells could be desirable for chronic lung infections as indicated by the successful use against lung *M. tuberculosis* infections; photodynamic therapy and antimicrobial peptides would be optimal for topical use, but not for systemic infections.; antibiofilm therapy would be associated against lung and prosthetic-related infections; and finally, and importantly, phage therapy and nanoparticles could be used to treat both local and systemic infections. 

All reviewed therapies are hopeful against multidrug resistant bacteria and show good results in vitro and in vivo. The advantages and disadvantages of these therapies are summarized in Table 2. However, to date only phage therapy and photodynamic therapy have been proven in patients against MAC. There is a general lack of data in most of the proposed alternative therapies to understand the underlying mechanism of action and the correct administration to the patient. Another relevant issue to take into account is the immune status of the patient receiving these therapies, which can make it difficult to elaborate a standard protocol for every single therapy against MAC infections. It seems reasonable that these therapies against MAC infections may be administered as personalized therapies, although the cost of this type of medicine is always higher.

The use of nanoparticles increases chemical stability of pharmacological agents and reduces side effects, making this a very attractive strategy against resistant bacteria. However, the scaling and quality control of nanoparticles is challenging and stability is particularly complex in the case of nanomaterials.

## Figures and Tables

**Table 1:** Use of antimicrobial peptides against *M. abscessus*.

Peptide	In Vitro/In Vivo	Material and Methods	Outcome	References
**Polydim-I**	In vitro and in vivo	Treatment of macrophages and mice infected with *M. abscessus*	Reduction of bacterial load by 40–50% in macrophages and 0.8 log in mice	[43]
**Polybia-MPII**	Ex vivo	Treatment of murine peritoneal macrophages infected with *M. abscessus* and cytotoxicity assays	Reduction of bacterial load by 80% at a concentration of 12.5 nM. Detection of high haemolytic activity	[44]
**NDBP-5.5**	In vitro and in vivo	Minimum inhibitory concentration determination and in vivo assay in mice compared to clarithromycin	Bacterial load reduction by 70% against clarithromycin action	[45]
**AP1, AP2, AP3-AP1, AP4**	In vitro	Minimum inhibitory concentration determination	Definition of minimum inhibitory concentration 1.6 to 50 µg/mL	[46]
**S5, S52, S6, S61, S62, S63, KLK, KLK1, KLK2, Pug-1, Pug-2, Pug-3 andPug-4**	In vitro	Minimum inhibitory concentration determination and cytotoxicity assays	(S61, S62, S63 and KLK1) providing MICs between 200–400 µg/mL. Cytotoxicity: S63 was the least toxic and KLK1 the most toxic	[47]
**RP557**	In vitro	Synergies in combination with clarithromycin, amikacin, cefoxitin and imipenem against *M. abscessus* in biofilm	Reduction of biofilm formation and determination of the interference of RP557 on bacterial growth	[48]
**NZX**	In vitro and in vivo	In vitro and in vivo assay in combination with antibiotics	Reduction of bacterial load	[49]
**ASU2056 and ASU2060**	In vitro	Definition of minimum inhibitory concentration	Minimal inhibitory concentrations of 32 and 8 μM.	[27]

**Table 2:** Advantages and disadvantages of alternative therapies against *M. abscessus*.

Therapeutic Strategy	Advantages	Disadvantages
**Antibiofilm**	Compounds of very diverse chemical nature. Improves sensitivity to different antimicrobials. Reduces the persistence of *M. abscessus* in the human organism.	They do not have antimicrobial action by themselves.
**Nanoparticles**	Allow a greater and better distribution and bioavailability of antimicrobials. Reduce toxicity of drugs with respect to their free form. Reduce side effects.	Scaling and quality control is challenging.
**Vaccines**	Stimulate immune status against *M. abscessus.* Reduce the severity of infections.	In general, there is a greater response against smooth strains than against rough strains. It is a non-curative and only a preventive alternative. Still needs to be properly validated.
**Antimicrobial peptides**	Very diverse origin, both natural and chemical synthesis. Easy to obtain and to test against *M. abscessus.* Show antimicrobial activity both on their own and in combination.	May be toxic to human cells. Not degraded in human serum.
**Host Modulation Therapy**	Regulation of local immune response. Direct or indirect antibacterial activity (secreting NO, antimicrobial peptides, autophagy). Repairing tissue damage. Accumulation at site of infection.	Potential side effects. Different biological functions of MSCs in different tissue sources and in vitro culture conditions. Treatment selected according to individual immune status. Different lifestyles of Mycobacteria′s host cells.
**Photodynamic therapy**	High antibacterial effectiveness. Broad antimicrobial spectrum. Low host damage and fewer side effects. No resistance induction to the therapy. Short treatment time. Little invasiveness.	Treatment effectiveness depends on suitable PS and light. Photosensitivity after treatment.
**Phage therapy**	Single phage treatment is possible. Specificity of action. Treatment of chronic and recurrent infections with no other clinical outcome. Easy administration. Can be improved by engineering.	Possible emergence of bacterial resistance. High costs for formulation and stabilization of pharmaceutical preparations. Neutralizing antibodies. Very few clinical trials.
